# Genomic Characterization of Novel Circular ssDNA Viruses from Insectivorous Bats in Southern Brazil

**DOI:** 10.1371/journal.pone.0118070

**Published:** 2015-02-17

**Authors:** Francisco Esmaile de Sales Lima, Samuel Paulo Cibulski, Helton Fernandes dos Santos, Thais Fumaco Teixeira, Ana Paula Muterle Varela, Paulo Michel Roehe, Eric Delwart, Ana Cláudia Franco

**Affiliations:** 1 Virology Laboratory, Department of Microbiology, Immunology and Parasitology, Institute of Basic Health Sciences, Federal University of Rio Grande do Sul (UFRGS), Porto Alegre, Rio Grande do Sul (RS), Brazil; 2 FEPAGRO Animal Health, Institute of Veterinary Research "Desidério Finamor" (IPVDF), Eldorado do Sul, Rio Grande do Sul (RS), Brazil; 3 Blood Systems Research Institute, San Francisco, California, United States of America; University of California, Department of Laboratory Medicine, San Francisco, California, United States of America; Institut Pasteur, FRANCE

## Abstract

*Circoviruses* are highly prevalent porcine and avian pathogens. In recent years, novel circular ssDNA genomes have recently been detected in a variety of fecal and environmental samples using deep sequencing approaches. In this study the identification of genomes of novel circoviruses and cycloviruses in feces of insectivorous bats is reported. Pan-reactive primers were used targeting the conserved *rep* region of circoviruses and cycloviruses to screen DNA bat fecal samples. Using this approach, partial *rep* sequences were detected which formed five phylogenetic groups distributed among the *Circovirus *and the recently proposed *Cyclovirus* genera of the *Circoviridae*. Further analysis using inverse PCR and Sanger sequencing led to the characterization of four new putative members of the family *Circoviridae* with genome size ranging from 1,608 to 1,790 nt, two inversely arranged ORFs, and canonical nonamer sequences atop a stem loop.

## Introduction

Viruses of the *Circoviridae* family are known to infect a wide range of vertebrates. The virions consist of naked nucleocapsids of about 20 nm in diameter, with a circular single stranded DNA (ssDNA) genome of approximately 2.0 kb [1]. They have an ambisense genome organization containing two major open reading frames (ORFs) inversely arranged, responsible for encoding the replicase (Rep) and capsid (Cap) proteins, and are separated by a 3’ intergenic region (IGR) between the stop codons and a 5’ IGR between the start codons [2]. Some circoviruses are major pathogens of pigs [3–5], e.g. porcine circovirus 2 (PCV2) which causes either asymptomatic infections or clearly apparent disease which may be responsible for significant economic losses [6–10]. In birds, avian circoviruses, within the genus *Gyrovirus*, have been identified in a broad range of avian species; one of them, chicken anemia virus (CAV), is a major cause of disease, associated to lymphoid depletion, immunosuppression and developmental abnormalities [11–15]. According to the document 2014.006a-gV from ICTV, there is a proposal of the *Gyrovirus* genus removal from *Circoviridae* to *Anelloviridae* family due to recent metagenomic studies on gyroviruses showing a very high sequence divergence when compared to other circoviruses members.

Recent metagenomic approaches, high-throughput sequencing techniques and degenerate PCRs have led to the identification of small circular DNA genomes in fecal samples of wild mammals, in insects as well as from environmental samples [2,16–18]. Some of the newly described circular genomes are similar to those of circoviruses, but phylogenetically different from the previously known avian and porcine circoviruses [18]. Their distinct nucleotide/amino acid composition and genome organization allowed authors to propose the creation of a new genus within the *Circoviridae*, which was named *Cyclovirus*. In comparison to members of the genus *Circovirus*, both *rep* and *cap* cycloviruses genes are smaller, with shorter or no 3’ IGR between the stop codons of the two major ORFs and a longer 5’ IGR between the start codons of the two major ORFs [2].

Sequences related to circoviruses have been identified based on the detection of the conserved Rep region involved in rolling circle replication (RCR) [19]. *Cyclovirus* genomes were detected in wild animal’s samples, human feces and cerebrospinal fluids; muscular tissues of farm animals such as chickens, cows, sheep, goats, and camels [20,21]. Currently, eight different species of cycloviruses have been detected in winged-insect populations highlighting they circulate in a wide host range possessing a high genetic diversity, as well [18–20,22–24].

So far, classification for the genus *Circovirus* considers circoviruses sharing >75% genome-wide nucleotide identity and >70% amino acid identity in the capsid (Cap) protein to the same species. Although, there are no species demarcation criteria for the genus *Cyclovirus*, the taxonomic classification for the family *Circoviridae* considers viruses sharing >60% in their Cap amino acid identity level as belonging to distinct genera [19].

In the present article, the detection of ssDNA genomes from bat fecal samples is reported. Genome segments were amplified by consensus/degenerate PCR. Whole genome sequencing and phylogenetic analyses of the sequences obtained revealed that four of the sequences represent viral genomes of new members of the family *Circoviridae*.

## Materials and Methods

### Ethics Statement

Permission for this work on protected bats was granted by Health Monitoring (CEVS—Centro Estadual de Vigilância em Saúde) of the Brazilian federal state of Rio Grande do Sul. The study did not involve any direct manipulations of bats and relied entirely on collection of fecal samples from the attic floor. All experiments were performed in compliance with the European Convention for the Protection of Vertebrate Animals Used for Experimental and Other Scientific Purposes (European Treaty Series—No. 170 revised 2005) and the procedures of the Brazilian College of Animal Experimentation (COBEA). It must be highlighted that we had the owner’s permission to access the attic for the purposes of this study. In case of future surveys in Porto Alegre, the Health Monitoring (CEVS) will be contacted to obtain the permissions.

### Sample collection and preparation

A maternity roost of bats was identified in the summer of 2012 in the attic of a private residence in the central area of the municipality of Porto Alegre, Rio Grande do Sul, Southern Brazil. The colony was estimated to harbor about 500 bat specimens of insectivorous bats of two species, velvety free tailed bats (*Molossus molossus*) and brazilian free tailed bats (*Tadarida brasiliensis*) [25]. Speciation was confirmed by DNA extraction from fecal pellets, amplification and sequencing of the mitochondrial cytochrome b (*cytb*) gene as described [26].

One hundred fecal samples were collected from the attic floor as follows: a plastic film was spread on the ground of the attic compartment and fresh droppings were collected with clean disposable forks in the following night. Each sample consisted of pool of 5 fecal droppings, which were immediately sent to the laboratory and stored at -80°C. The samples were then thawed, resuspended and in 1 mL of Hank’s balanced salt solution (HBSS), vortexed and centrifuged at 10.000 x *g* for 5 min. The supernatants were then transferred to fresh tubes, filtered through 0.45 μm pore-size syringe filters (Fisher Scientific, Pittsburgh, PA) and submitted to DNA extraction.

### DNA extraction, PCR and sequencing

Total fecal DNA was extracted from 400 μL of the supernatants (above) with phenol-chloroform (Invitrogen) [27]. The extracted DNA was eluted in 50 μL of TE (Tris-hydrochloride buffer, pH 8.0, containing 1.0 mM EDTA), treated with 20 μg/mL of RNase A (Invitrogen) and stored at -80°C. Subsequently, samples were submitted to amplification in a nested-PCR targeting the *rep* gene of circoviruses/cycloviruses with the following degenerate primers: CV-F1 (5´-GGIAYICCICAYYTICARGG-3´), CV-R1 (5`-AWCCAICCRTARAARTCRTC-3`), CV-F2 (5´-GGIAYICCICAYYTICARGGITT-3´), and CV-R2 (5´-TGYTGYTCRTAICCRTCCCACCA-3´) [2]. Briefly, the nested PCR was performed as follows: the first reaction was performed in a 25 μL volume containing 20 to 50 ng of sample DNA 1 mM MgCl_2_, 0.2 μM of each primer (CV-F1 and CV-R1), 1.5 U Taq DNA polymerase (Invitrogen), 10% PCR buffer and 0.6 mM dNTPs. The cycling conditions were: 5 min at 95°C; 40 cycles of 1 min at 95°C, 1 min at 52°C, 1 min at 72°C and a final incubation at 72°C for 10 min. For the second (nested) reaction, the 25 μL mix components were: 1 μL of the 1^st^ reaction product, 1 mM MgCl_2_, 0.2 μM of each primer (CV-F2 and CV-R2), 1.5 U Taq DNA polymerase (Invitrogen), 10% PCR buffer and 0.6 mM dNTPs. The cycling conditions were: 5 min at 95°C; 40 cycles of 1 min at 95°C, 1 min at 56°C, 1 min at 72°C, and a final incubation at 72°C for 10 min. Products with a size of approximately 400 bp were purified and directly sequenced using primer CV-R2. To confirm the sequences, each product was sequenced three times. Samples were sequenced with the Big Dye Terminator Cycle Sequencing Ready Reaction (Applied Biosystems, UK) in an ABI-PRISM 3100 Genetic Analyzer (ABI, Foster City, CA), according to the protocol of the manufacturer. Sequences similar to the *rep* gene sequences of circovirus-like-genomes were aligned for designing of new sets of primers to perform the inverse PCR (iPCR). The iPCR were carried out in a 25 μL reaction mixture optimized with Platinum Taq Hi-Fi (Invitrogen™) (cycling conditions can be informed upon request) and the primer sequences as follows: Circo1_F (5’-CTTTCTCCCAGTTAATTCTCC-3’), Circo1_R (5’-GAGCAAGTGGAACAGGTAAAT-3’), Circo2_F (5’-TCAAAGCGTGCACTTGA-3´), Circo2_R (5’-ACAGGATTGTAGATCAGTACGT-3’), Circo5_F (5’-GAGATGCAAGAATGGATTGC-3’), Circo5_R (5’-GCAATATTTGCTTGCCGC-3’), Circo6_F (5’-GAGAGTATCAACGCAGAAAG-3’), Circo6_R (5’-CTTTGATAAATTAGCGAACC-3’), Circo7_F (5’-AGAGGCTCAACAAATAGACGC-3’), Circo7_R (5’-TGCTTTTTGATGGTACTGAA-3’). Standard precautions were taken to avoid contamination and negative controls were added to each batch of reactions. Five microliters of the PCR products were electrophoresed in 0.7% agarose gels and the products visualized on UV light after staining with ethidium bromide. The amplicons corresponding to the sizes ranging from 1–2 kb were purified and cloned into pCR 2.1-TOPO cloning kit (Invitrogen™). Three insert-containing plasmids of each clone were sequenced with M13 forward and reverse primers as described above. The full-length sequence of genomes was constructed by “genome walking” using the Geneious software (version 7.1.3).

### Gene identification and phylogenetic analysis

Identification of putative ORFs was made with aid of ORF Finder (NCBI; http://www.ncbi.nlm.nih.gov/gorf/gorf.html). Sequence analyses were performed with the BLASTX software (http://www.ncbi.nlm.nih.gov/blast/). Nucleotide sequences were aligned and compared to sequences of human, animal and sewage-associated members of the *Circoviridae* available at GenBank database using ClustalW [28]. The alignments were optimized with the BioEdit Sequence Alignment Editor Program version 7.0.9 [29]. The hairpin and stem-loop structures were identified in Mfold [30]. Phylogenetic analysis was carried out in MEGA5 [31]. The confidence of each branch in the phylogeny was estimated with bootstrap values calculated from 2000 replicates. For the purpose of this work, the samples were named Bat Circovirus Porto Alegre (BatCV POA), followed by the cluster number to which each one was assigned.

## Results

### Molecular detection and genetic diversity of circovirus-like rep sequences in feces of insectivorous bats

**Fig 1:**
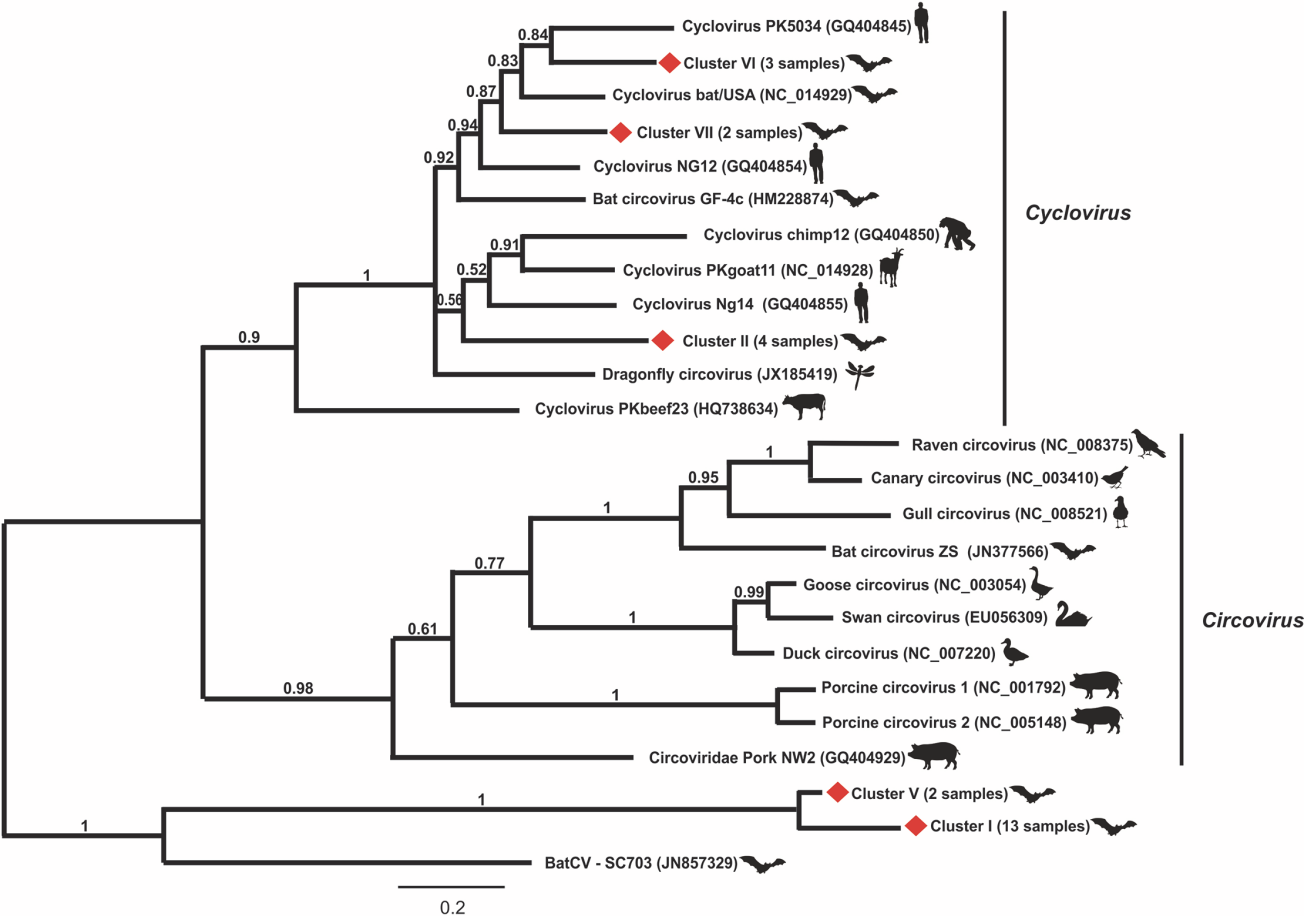
Phylogenetic analysis of partial REP protein sequences obtained from pooled bat fecal samples compared to representative members of *Circoviridae* family. The twenty-four REP sequences of bat-sourced circoviruses formed five clusters, labelled by red diamonds from I–VII. The evolutionary history was inferred by using the maximum likelihood method based on the Poisson correction model. Evolutionary analyses were conducted in MEGA5 [31].

Amplicons with the expected size (about 400 bp) were obtained from 24 out of the 100 (24%) fecal samples screened. The amplified DNA was direct sequenced. The nucleotide sequences corresponding to part of the *rep* gene were determined and submitted to GenBank (KM401658-KM401681). BLASTX analysis showed that these partial *rep* sequences have an amino acid identity of 10–76% with those of known circoviruses and 87–100% among themselves. A phylogenetic tree was constructed based on the alignment of the deduced amino acid sequences herein detected with those of the representative *Circovirus* and *Cyclovirus* sequences (Fig. 1). As shown in the tree, it was observed the arrangement of five main groups with clusters II (4 sequences), VI (3 sequences) and VII (2 sequences) falling into the clade of cycloviruses, in contrast to clusters I (13 sequences) and V (2 sequences) that formed distinct and distant groups from those formed by circoviruses and cycloviruses. The arbitrary division of these sequences in clusters was carried out to analyze their genomic features, assuming that according to the criteria used for *Circovirus* diversity analysis, distinct species comprising more than >20% sequence divergence are considered to be classified as an individual viral [32]. According to this, we could infer the detection of five potential new species from bat samples (3 cycloviruses and 2 circoviruses).

### Genomic characterization of the new putative circovirus-like sequences in insectivorous bats

Two full-length circular ssDNA genomes of 1,755 and 1,790 nt (named BatCV POA/2012/II and BatCV POA/2012/VI, respectively) and two nearly complete circular DNA genomes of 1,720 and 1,750 nt (named BatCV POA/2012/I and BatCV POA/2012/V) were identified (GenBank accession numbers: KM382269-KM382272). It was not possible to amplify the genome comprising those of cluster VII.

The impossibility to achieve the complete sequencing of virus DNA from clusters I and V was probably due to the high GC-rich content present in the 3´ IGR GC region, even though attempts on PCR amplification before sequencing were made without much success by varying the concentrations of DMSO and/or in the presence of 50% 7-deaza-GTP and 50% dGTP (New England Biolabs), as performed by Rijsewijk et al. [33].

**Fig 2:**
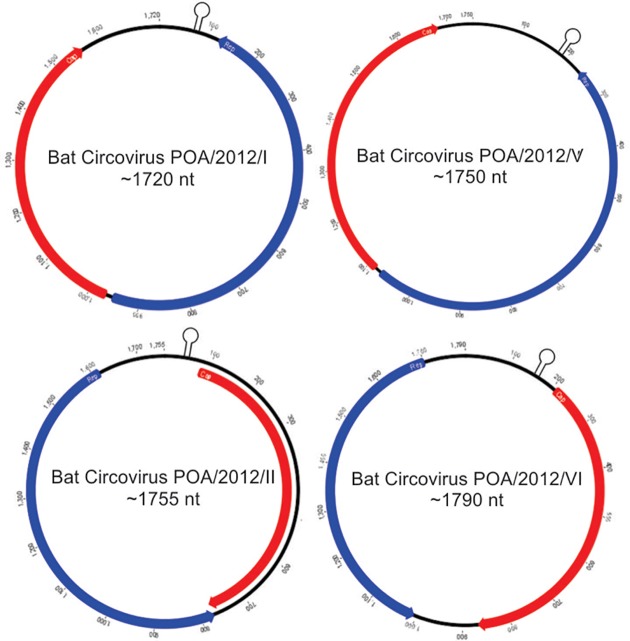
Predicted genome organization of the four circular ssDNA sequences with the locations of the potential stem-loop structures recovered from bat feces in Southern Brazil. The two inversely arranged ORFs responsible for encoding the putatives replication associated protein (REP) and capsid protein (CAP) are shown in blue and red boxes, respectively.

The predicted two ORFs, *rep* and *cap*, are present and inversely arranged in all sequences as shown in Fig. 2. The predicted CAP protein sequences consist of 197–231 amino acids and share an amino acid identity of 24–76% with the known cycloviruses/circoviruses and 15.5–88.8% among themselves (Tables 1 and 2). The predicted REP protein sequences ranged from 232 to 280 amino acid and have an amino acid identity ranging from 9.2–44.4% among themselves (Tables 1 and 2).

**Table 1:** Main features of BatCV POA genomes.

	Genome (nt*)	Cap				Rep			
		Position (nt)	Strand	Size (nt)	Size (aa)	Position (nt)	Strand	Size (nt)	Size (aa)
BatCV POA/I	1720	967–1560	+	594	197	953–120	-	834	277
BatCV POA/II	1755	1676–981	-	696	231	142–984	+	843	280
BatCV POA/V	1750	643–47	+	597	198	657–1490	-	834	277
BatCV POA/VII	1790	1583–924	-	660	219	88–786	+	699	232

*The nucleotide sizes of partial genomes I and V (GenBank accession numbers KM382269 and KM382271, respectively) were estimated by ImageJ. Genomes II (KM382270) and VI (KM38272) were completely sequenced.

**Table 2:** Pairwise comparison of the BatCVs POA I, II, V and VI based on amino acid identities (%) shared by CAP and REP proteins.

	Cap protein				Rep protein			
Genomes	BatCV POA/I	BatCV POA/II	BatCV POA/V	BatCV POA/VI	BatCV POA/I	BatCV POA/II	BatCV POA/V	BatCV POA/VI
BatCV POA/I	-	15.5	88.8	17.5	-	30.4	9.2	21.4
BatCV POA/II	15.5	-	15.9	33.9	30.4	-	31.8	44.4
BatCV POA/V	88.8	15.9	-	17.5	9.2	31.8	-	20.7
BatCV POA/VI	17.5	33.9	17.5	-	21.4	44.4	20.7	-

Stem-loop structures were found in all 4 bat circular genomes. They have a conserved nonanucleotide motif located at the 5’ IGR (NANTATTAC) and are considered to be responsible for initiating the rolling-cycle replication of circoviruses [18,34]. As shown in Table 3, all four BatCV POA also contain a conserved nonamer sequence in the loop region of the 5’ IGR, different from the conserved *Cyclovirus* and *Circovirus* nonanucleotide motif sequence, but similar to the loop motif of cycloviruses found on bat, human and chimpanzee feces (BatCV POA II, V, VI) and slightly modified from those of *Cyclovirus* and *Circovirus* (BatCV POA I) [2,17,18,20].

**Table 3:** Organization and genomic features of the potential stem-loop structures found in the four BatCV/POA genomes.

Genome	Putative stem-loop structure	Hairpin length	Loop length	Nonamer sequence
BatCV POA/I	5’ gagttttgtGCACAGTATTACCacaaaactc 3’	31	13	CAGTATTAC
BatCV POA/II	5’ cgaagtgacgGTTAGTATTACCcgtcacttcg 3’	32	12	TAGTATTAC
BatCV POA/V	5’ gagttttgtGCATAGTATTACCacaaaactc 3’	31	13	TAGTATTAC
BatCV POA/VI	5’ cgaagtcggGTATAGTATTACCccgacttcg 3’	31	13	TAGTATTAC

**Fig 3:**
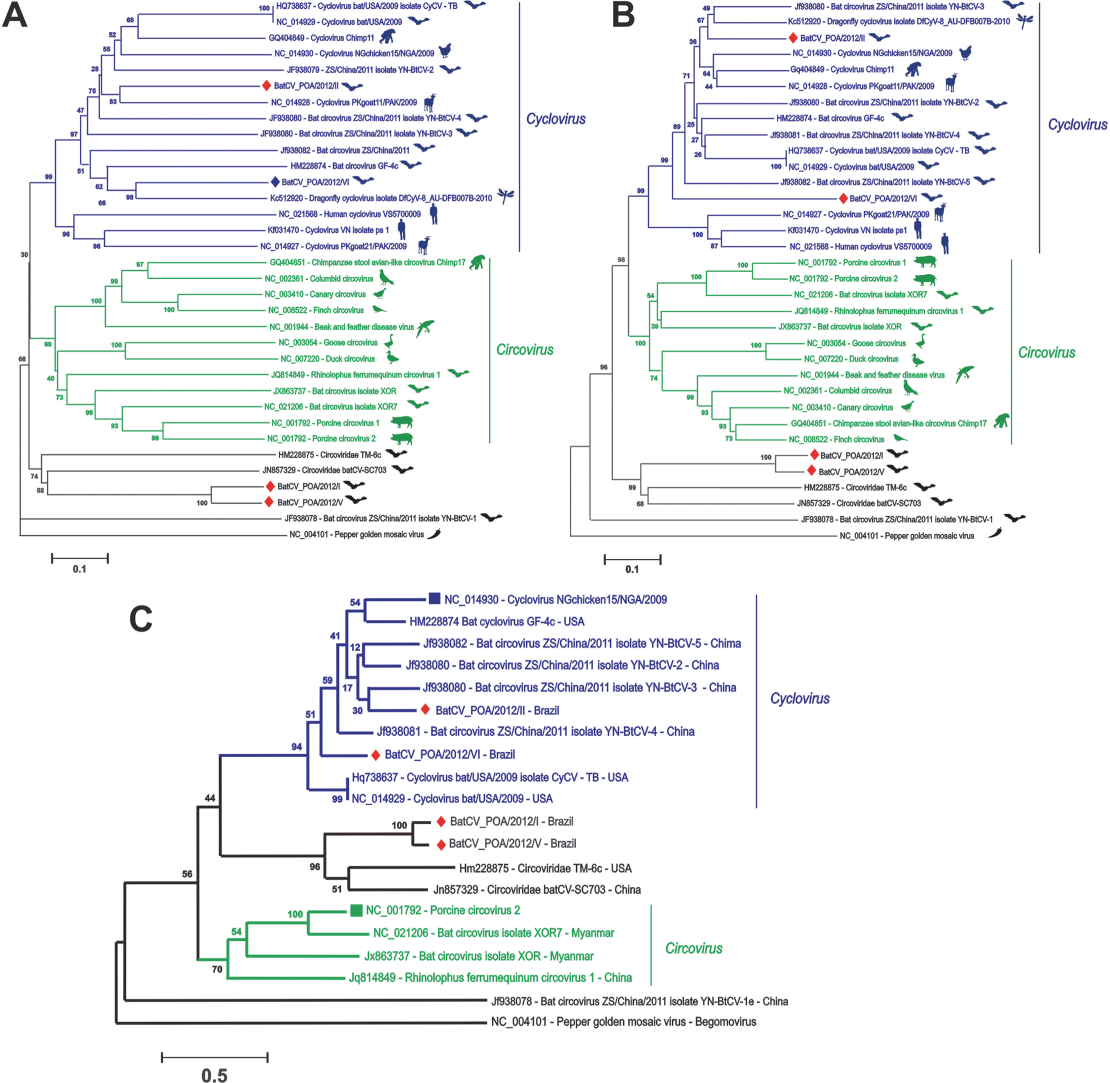
Phylogenetic analysis of the complete CAP (A), REP (B) and complete genomes (C) from *Circovirus* and *Circovirus*-like genomes identified in mammals, birds and insects. Host denomination is demonstrated after each retrieved sequence from GenBank with their accession numbers and clustered along, according to genus classification. The sequences identified in bat feces from Southern Brazil are labeled by red diamonds. Evolutionary analyses were conducted in MEGA5 [31].

The predicted protein sequences encoded by ORF2 (*cap*) and ORF1 (*rep*) of BatCV I-VI genomes were used for phylogenetic analysis with representative and recently discovered circoviruses/cycloviruses; Pepper golden mosaic virus was used as outgroup, as they are somewhat related to other members in the *Circoviridae* family (Fig. 3A, 3B and 3C). As shown in the trees, BatCV POA/2012/II and VI fell into the cyclovirus clade already identified in chickens, chimps, bats, goats, humans and dragonflies [2,17,18,20,22]. When analyzing the *cap*-encoding region (Fig. 3A), BatCV POA/2012/II was related to a *Cyclovirus* detected in muscle tissues of a goat from Pakistan through degenerate/consensus PCR [20], and BatCV POA/2012/VI was more related to dragonfly *Cyclovirus* detected through viral metagenomics [22]. However, when analyzing both genomes according to the conserved *rep*-encoding region, it was observed that they formed a monophyletic clade (Fig. 3B). On the other hand, BatCV POA/2012/I and V fell outside the *Circovirus* and *Cyclovirus* clades, not yet related to any genus of *Circoviridae* family along with Bat circovirus-like virus TM6 and batCV-SC703 [17,18]. This situation was confirmed based on the alignments of the whole genomes, producing a similar tree topology (see Fig. 3C). These sequences are closer to sequences detected in guano and fecal samples collected from bats in the United States and China through metagenomic approaches, suggesting that these viruses have the same host origin, likely from bats [17,18]. However, currently, no classification has been fully considered to these sequences.

## Discussion

In this work we report the discovery of 4 novel circular ssDNA genomic sequences from insectivorous bats feces from Brazil. In the recent years, many genomes of circoviruses, cycloviruses and *rep*-containing circular DNA viruses have been characterized in mammals, birds, insects and environmental samples [19] bringing to light a high level of genetic diversity among these viruses [19,35]. According to our results, two genomes belong to genus *Cyclovirus* (BatCV POA II and VI). These genomes are organized and contain two major ORFs in opposite directions, presenting in their 5’ IGR of the *rep* ORF the cyclovirus-conserved nonanucleotide motif (5’-TAATACTAT-3’) in their loop region (Table 3). BatCV POA I and V present their *cap* located in the positive strand and the larger *rep* located on the minus strand, as expected for circoviruses, but this pattern was not present in BatCV POA II and VI, as shown in Table 1. The phylogenetic analysis constructed based on the alignments of the complete REP and CAP protein confirms that BatCV POA/II and VI cluster into the genus *Cyclovirus* along with the Chinese cycloviruses sequences clade detected in bat feces [18] and sharing less than 65% of identity at the CAP/REP amino acid level. BatCV POA I and V had a low amino acid identity with CAP (<20%) and REP (<10%) sequences of two other sequences detected in bat feces in this study with known circoviruses/cycloviruses (Table 2). Consequently, they formed a distinct clade along with other bat-sourced sequences, expanding the view of diversity in these new ssDNA viruses that are divergent enough at the sequence level that they could very likely be part of a different genus.

In our study, we detected *Cyclovirus* and *Circovirus* related sequences at a frequency of 24% in the examined samples. However, due to methodological limitations, restriction in location and variety of bat species, we were not able to extrapolate our results to epidemiological data (such as incidence and prevalence) or to which bat species the ssDNA positive samples belonged. As performed by Ge et al. in China [36], further investigation is needed to determine the prevalence of circoviruses in other Brazilian bat species. Nevertheless, it becomes clear that such study is worthy to understand the great diversity of circoviruses found worldwide.

Our study was based on the phylogenetic analysis and comparison to the sequences recovered. The finding of known insect viruses in bat feces simply reflects the diet of these insectivorous bats, which play an important role on predating insects. Viral DNA detection in bat feces does not allow one to differentiate between viral replication in bats or simple passage through the digestive track from ingested food [20,35].

To date, few members of the *Circovirus* genera can be related to severe clinical conditions in animals, with the exception of PCV2 and some of the avian circoviruses [5]. Even with the recent discovery of many cycloviruses, circoviruses-like or rep-like sequences in a variety of mammals tissues and feces, including humans fecal samples [20,36,37], there is no syndrome yet associated with these viruses. Nevertheless, a recent identification of a new *Cyclovirus* from Vietnamese and Malawi patients with acute central nervous system infection of unknown etiology raises the possibility of disease association, yet to be proven [38,39], although possibly with limited geographic distribution [38].

In this work, two more circular DNA genomes were characterized which did not fall within the circo/cycloviruses clade grouping instead distantly with TM6 and batCV-SC703 [17,18] both also from bat feces. These new genomes have in common the presence in the Rep N-terminus of the same motifs associated with rolling circle replication (FTLNN, TPHLQGY) and dNTP-binding (GXGKS), as well as the conserved identified in the carboxy half of Rep amino acid motifs associated with 2C helicase function (WWDGY and DRYP) [19]. The N-terminal regions related to Cap proteins of BatCV POA I and V are highly basic and arginine-rich, as is typical for circoviruses capsid proteins with arginine residues ranging from 36%-42% (Genome I and V, respectively) along the first 50 aa, in contrast to TM6 (28%) and SC703 (26%). They are also distinguishable based on their CAP and REP sizes (data not shown), as well as on the low amino acid level for both proteins, as the percentage of amino acid identity of BatCV POA I and V shows a REP identity <45% and <35% for CAP identity in relation to TM6 and SC703. Based on these genomes characteristics, even though they are clustered in a separate clade, not yet characterized, they are new viral species. Upon the discovery of other sequences grouping along with these genomes, it will be of interest to propose the creation of a new genus within *Circoviridae* by the International Committee on Taxonomy of Viruses (ICTV).

Here we report the detection of four novel circular ssDNAs from bat feces after whole-genome characterization within the family *Circoviridae*. So far, it is not clear if these new ssDNA detected have some important role on pathogenesis. In addition to bioinformatics analysis, future investigations must include attempts in virus isolation to confirm host origin, which will give some light to better understand the relationships between these circular DNA viruses and bats.
